# Enchainment of the Coefficient of Structural Quality of Elements in Compression and Bending by Combined Reinforcement of Concrete with Polymer Composite Bars and Dispersed Fiber

**DOI:** 10.3390/polym13244347

**Published:** 2021-12-12

**Authors:** Sergey A. Stel’makh, Evgenii M. Shcherban’, Alexey Beskopylny, Levon R. Mailyan, Besarion Meskhi, Natal’ya Dotsenko

**Affiliations:** 1 Department of Engineering Geology, Bases, and Foundations, Don State Technical University, 344003 Rostov-on-Don, Russia; sergej.stelmax@mail.ru (S.A.S.); au-geen@mail.ru (E.M.S.); 2 Department of Transport Systems, Faculty of Roads and Transport Systems, Don State Technical University, 344003 Rostov-on-Don, Russia; 3 Department of Roads, Don State Technical University, 344003 Rostov-on-Don, Russia; lrm@aaanet.ru; 4 Department of Life Safety and Environmental Protection, Faculty of Life Safety and Environmental Engineering, Don State Technical University, 344003 Rostov-on-Don, Russia; reception@donstu.ru; 5 Department of Technological Engineering and Expertise in the Construction Industry, Don State Technical University, 344003 Rostov-on-Don, Russia; natalya_1998_dotsenko@mail.ru

**Keywords:** polymer composite reinforcement, dispersed fiber, concrete, coefficient of structural quality, glass fiber, compressive strength, tensile strength

## Abstract

Polymer composite reinforcement (PCR) and its use to produce high-quality concrete with the right design and technological and formulation solutions can demonstrate the results obtained with the steel rebars. This article discusses the synergistic effect from the combined reinforcement of concrete with traditional polymer rods and dispersed fiber, which, as a result, lead to an increase in strength and deformation characteristics and an improvement in the performance of compressed and bent structural elements. The synergistic effect of the joint work of polymer rods and dispersed reinforcement is considered in the context of relative indicators (structural quality factor CSQ), showing the relationship between strength characteristics and concrete density. The behavior of glass fiber in a cement matrix and the nature of its deformation during fracture were studied by scanning electron microscopy. It is shown that the use of PCR and dispersed reinforcement makes it possible to increase the strength characteristics of concrete in bending. In quantitative terms, the achieved results demonstrated that the CSQ values of a beam reinforced with a PCR frame with the addition of glass fiber were 3.4 times higher compared to the CSQ of a beam reinforced with steel reinforcement frames. In addition, for a beam reinforced with a PCR frame with no fiber addition, the CSQ values were three times higher.

## 1. Introduction

Currently, construction quality requirements are higher due to the increase in the number of stories of buildings and high-rise and large-span unique objects. Consequently, the requirements for materials and structures of buildings being erected have significantly increased [1,2,3]. New types of materials have entered the building materials and ready-made structures market related to the sustainable reduction and compensation of CO_2_ emissions [4], the need to reduce the weight of structures [5], to increase the economic efficiency of construction [6], and the development of lightweight and, at the same time, durable building materials [7]. The leading role in this is given to composite polymer building materials.

In recent years, considering the development of multi-story construction, the percentage of the use of the so-called polymer composite reinforcement has increased [8], which was designed by many scientists to displace over time the classical steel reinforcement that provides rod reinforcement of traditional reinforced concrete. In turn, reinforced concrete, as a building material tested over many decades, is undoubtedly a material with many advantages. Still, the disadvantages of this material in recent years are apparent. First, it contributes, of course, a significant weight to the resulting structures and, consequently, the complexity of their construction. Second, the steel reinforcement leads to high construction costs and, ultimately, colossal resource, energy, and labor costs in constructing buildings and structures.

Steel reinforcement does not fully implement its strength potential and is limited by the mechanical characteristics of concrete [9,10]. It should also be added that there is a danger that has been repeatedly described in the regulatory and technical literature; in expert cases, these are prestressed reinforced concrete elements in which steel reinforcement is an element posing a danger to builders and operators.

Thus, polymer composite reinforcement is an efficient solution. Many scientists dealing with issues and problems of construction are in search of optimal design solutions, that is, the number, size, and cross-section of rods located in the concrete body [11,12,13,14]. The issues of joint operation of polymer composite reinforcement with cement stone remain unsolved. Structural changes in fiber-reinforced concrete during the destruction and many other aspects that cannot and in a short time remain poorly studied, and a large number of experimental and theoretical studies are required.

In this regard, an urgent topic for research is the development of new structural, theoretical, and technological solutions for obtaining effective polymer composite reinforced concrete structures for new types of buildings and structures.

Significant strengthening of concrete structures [15,16,17,18] can be obtained using adhesive systems made of carbon fiber-reinforced polymer (CFRP). The authors of [19] presented a new CFRP reinforcement system with a high utilization factor. It was shown that the regimes of average deflection and yield were improved by 220 and 300%, respectively, and brittle fracture regimes were also prevented in which the collapse of concrete in compression and fracture of the anchor occurred almost simultaneously.

Polymer composites have excellent corrosion resistance and strength properties. However, their long-term reliability can be reduced by creep and fatigue [20]. Nevertheless, numerical analysis and experimental research show excellent properties of composite materials under complex loading and higher temperatures. An experimental study [21] confirmed an increase in the fatigue strength of a reinforced CFRP slab, 32% higher than the design operating load. The problem of fatigue accumulation of defects in fiber-reinforced concrete structures supporting underground workings and tunnel lining from dynamic loading was studied in [22]. Numerical modeling of composite concrete was carried out in the Abaqus software package. It was shown that the presence of a sufficient amount of fiber in the composition of concrete made it possible to avoid damage to the tunnel lining under dynamic loading completely. An amount of fiber less than 7 kg/m^3^, namely, 3 and 5 kg/m^3^, reduced the level of damage to the lining structure after seismic impact.

The aim of [23] was to investigate concrete-coated, hollow-core slabs to understand their effect on the bending of composite steel girders, considering that the depth of the hollow-core slabs is more significant than that recommended by SCI P401. Furthermore, the constructive solutions proposed by the authors showed similar resistance to external loads. Therefore, the same strength can be obtained for a smaller amount of transverse reinforcement [23].

To improve flexural strength and impact characteristics, in [24], thin panels of ultra-high-performance steel fiber-reinforced concrete were additionally reinforced with outer layers of thermoplastic composites with the addition of continuous fibers. Under quasi-static loading, both types of thermoplastic composite reinforcement resulted in a 150–180% increase in both maximum load and toughness.

Bonded reinforcement is commonly used in concrete in civil engineering to increase the load-bearing capacity of a structure or to minimize the negative effects of long-term operation and possible defects. The quality of the adhesive bond between the reinforced structure and steel or composite elements is essential for effective reinforcement. In [25], a method was developed for detecting bond defects in adhesive joints between concrete beams and steel slabs using the modal analysis method. The results showed that the integrated modal analysis and wavelet transform could be successfully applied to determine the exact shape and position of delamination in the adhesive joints of composite beams.

Reinforced concrete beams made of a fine-grained fiber composite with the addition of steel fiber in the amount of 1.2% of the composite volume were studied in [26]. The results of the shear strength of reinforced concrete beams made of fine-grained fiber composite, bent under the action of a shear force, as well as the forces of cracking, causing the appearance of the first diagonal crack, were analyzed. The tests and analyses carried out have shown that the developed new fiber composite can be successfully used to manufacture building elements in terms of shear strength.

In [27], the emphasis was mainly on in-depth analysis of the role of polymer fibers in preventing the brittle nature of ultra-high-performance concrete (UHPC) cracking. The thermal mismatch between the embedded fibers and the matrix was critical to obtaining an interconnected network of fractures in the matrix. The fracture network was responsible for increasing the permeability, thereby reducing the susceptibility to brittle fracturing of the UHPC [27].

The authors of [28] investigated the behavior of a glass fiber-reinforced polymer (GFRP) reinforcing bar in an alkaline environment (concrete) for 0, 1, 2, 3, 4, 6, 14, and 24 months at 60 °C to assess its durability in a concrete structure. The moisture absorption of the reinforcement was only 0.76%. It is noted that the properties under study practically did not change during the exposure period from 1 month to 24 months. The design axial tensile strength and modulus of elasticity of the reinforcement were retained at 100% after 24 months in concrete. It was found that water absorption is the main factor affecting fiberglass reinforcement’s thermal and mechanical properties [28].

In [29], a comparative study of steel reinforcement and fiberglass reinforcement strength under tensile, compressive, and bending forces was carried out using LS-DYNA. The numerical analysis results showed that the stresses arising under tensile and compressive loads were 25 and 37.25% higher for fiberglass reinforcement than steel but within safe limits. By analogy, the deformation under tensile and compressive loads for fiberglass reinforcement was 62.16 and 87.12%, respectively, higher than steel. However, the load-bearing capacity of fiberglass reinforcement was almost the same as that of steel. Since both materials showed no signs of damage below 40 kN, fiberglass reinforcement could be used as a replacement material instead of steel in roof supports and columns in underground structures [29].

In the study [30], experimental and analytical work was carried out to analyze the behavior of fiberglass reinforcement under eccentric loads in comparison with traditional steel reinforcement in self-compacting concrete columns. GRP columns have approximately 24% lower load-bearing capacity than steel-reinforced columns. The analysis of the results showed good agreement with the experimental results for columns reinforced with steel. In contrast, columns reinforced with fiberglass show a noticeable scatter in the values of the studied characteristics [30].

An overview of classical and modern developments from fiberglass in strengthening and restoring civil engineering objects was provided in [31]. The results of experimental, numerical, and analytical studies related to the integration of FRP into buildings in addition to other structures are presented. The discussion highlights the performance of FRP (including binder additives) under extreme conditions such as elevated temperatures, salty environments, and freeze–thaw cycles. Several constraints, problems, and research needs associated with the successful, sustainable, and reliable implementation of FRP in civil engineering are highlighted and analyzed [31].

From the review above, it can be seen that only pure reinforcement types were compared, without any mixing or combination in the structural aspect. That is, the reinforced elements were compared only with steel rods or with dispersed fiber. Therefore, in connection with the development and formulation of a working hypothesis, we assume the so-called synergistic effect arising as a result of combined reinforcement with rods and fibers, which, as a result, will lead to an increase in many characteristics and an improvement in the operation of the obtained compressed and bent elements. Furthermore, in the above works, the authors investigated the absolute indicators: the strength of concrete under various types of stress–strain states, deformability of concrete in a stress–strain state. However, the issue of relative indicators, that is, the ratio of strength and density characteristics of the obtained concrete or reinforced concrete elements, has not been studied.

Thus, from the literature review and analysis, at least two scientific research deficiencies devoted to concrete enchainment issues with polymer composite reinforcement can be seen. Deficiencies are expressed in constructive and technological aspects.

In the constructive aspect, only certain types of reinforcement were previously compared without combining with others. In this sense, the combination of polymer reinforcement bars with fiber seems to be an urgent scientific task.

In the technological aspect, the absolute indicators were previously investigated: the strength and deformability of concrete under various types of stress–strain states. However, relative indicators have not been studied, that is, the ratio of strength and density characteristics of the resulting concrete or reinforced concrete elements. Based on this, the study’s goal was to obtain new theoretical knowledge about the work at the microlevel of the cement matrix and fibers and develop ideas about the operation of compressed and stretched elements with an increased coefficient of structural quality made of concrete, combined with polymer composite reinforcement and dispersed fiber. The study’s objectives will be to investigate using SEM analysis the nature of the contact zone of the cement matrix and fibers and directly fibers during destruction to determine the quantitative characteristics of the joint work of a reinforced concrete element at the micro and macro levels, their theoretical justification, and experimental confirmation.

## 2. Materials and Methods

The rational composition of concrete, the scheme, percentage of reinforcement with rods, and the rate of fiber reinforcement were adopted following [22,23,24,25,26,27,28,29,30,31].

To manufacture the columns, Portland cement of the PC 500 D0 brand (Oskolcement OJSC, Stary Oskol, cement plant site, Russia) was used as a binder. Table 1 shows Portland cement’s physical and mechanical characteristics, and Table 2 shows Portland cement’s mineralogical and chemical composition.

Natural crushed stone from quartzite rocks (Yug-Nerud, Pavlovsk, Voronezh Region, Russia) was used as a large, dense aggregate. The physical and mechanical characteristics of the coarse aggregate are presented in Table 3.

Quartz sand was used as a fine aggregate (Yuzhny GOK, Aksai, Rostov Region, Russia). The physical and mechanical characteristics of the fine aggregate are presented in Table 4.

For the manufacture of reinforcing cages, polymer composite reinforcement PCR (Yaroslavl Composites Plant, Yaroslavl, Russia) and steel reinforcement class A400 with a diameter of 6 mm (“Tyazhpromarmatura”, Aleksin, Russia) were used. Comparative characteristics of metal and composite reinforcement are presented in Table 5.

Glass fiber (“Armplast“, Nizhny Novgorod, Russia) treated with surfactant was used as dispersed reinforcement; the physical and mechanical characteristics are presented in Table 6.

As a control composition, heavy concrete was designed on dense aggregates of class B30 with the required workability grade P1 (cone draft 1–4 cm). The parameters of the composition of the concrete mixture obtained as a result of calculations are reflected in Table 7.

In total, three series of prototypes were made. Each series included two samples with dimensions of 600 mm × 150 mm × 150 mm. In addition, to control the strength of concrete, cubes were made in parallel with each series of pieces with a size of 10 mm × 10 mm × 10 mm (3 pcs.), and prism samples with a length of 150 mm × 150 mm × 150 mm (3 pcs.) were made to determine the ultimate deformations during axial compression and tension. The first series of pieces included two samples reinforced with Ø6 A400 metal reinforcement. The second series included two samples reinforced with Ø6 polymer composite reinforcement. The third series also included two samples reinforced with Ø6 polymer composite reinforcement with additional dispersed glass fiber reinforcement. Each first sample in the series was tested for short-term central compression, and every second sample in the series was tested for bending.

The preparation of the concrete mixture for the prototypes was carried out following the previously established procedure [2,33]. For the preparation of the fiber-reinforced concrete mixture, considering that in the technology of preparation of fiber-reinforced concrete the most crucial moment is the inclusion of fiber into the concrete mixture to ensure its uniform distribution throughout the volume, the method of forced mixing was applied according to separate technology. First, Portland cement was loaded into the concrete mixing plant, then fiber was sequentially introduced; after uniform distribution of glass fiber throughout the entire volume of Portland cement, coarse and fine aggregates were introduced, then all components of the mixture were mixed in dry form, followed by the introduction of mixing water.

In this study, we used:

- Technological equipment: laboratory concrete mixer BL-10 (LLC “ZZBO”, Zlatoust, Russia); laboratory vibrating platform SMZh-539-220A (LLC “IMASH”, Armavir, Russia);
- Testing equipment: hydraulic press PGI-500 (OOO NPK TEKHMASH, Neftekamsk, Russia) with a measurement error of ±1%; tensile testing machine R-50 (LLC “IMASH”, Armavir, Russia) with a measurement error of ±1%;
- Measuring instruments (NPO LABORKOMPLEKT, Moscow, Russia): measuring metal ruler 1000 mm with a measurement error of ±0.5 mm; laboratory scales with a measurement error of ±0.05%; device for measuring deviations from the plane NPL-1; device for measuring deviations from perpendicularity NPR-1.

For the manufacture of prototypes, standard forms of the FP-150 brand (NPO LABORKOMPLEKT, Moscow, Russia) were used. Compaction of the fiber-reinforced concrete mixture during the formation of the samples was carried out on a laboratory vibrating platform SMZH-539-220A with mechanical fastening, the vibration time averaged 60–90 s. The next day after molding, the samples were stripped and placed in a standard hardening chamber for 28 days until the design strength was achieved.

**Figure 1:**
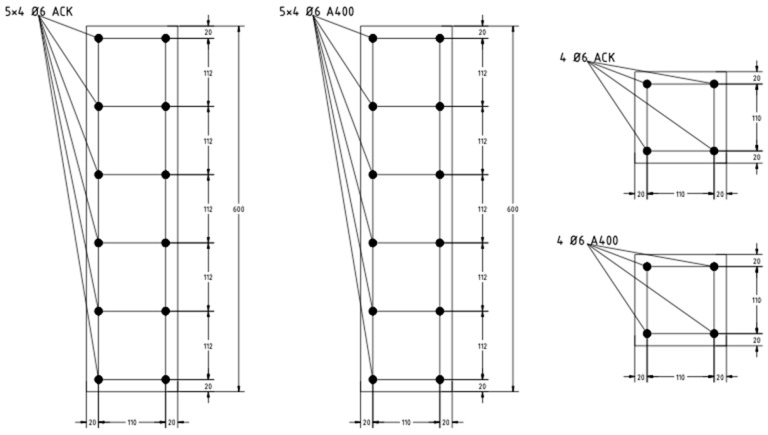
Scheme of the reinforcement of prototypes.

**Figure 2:**
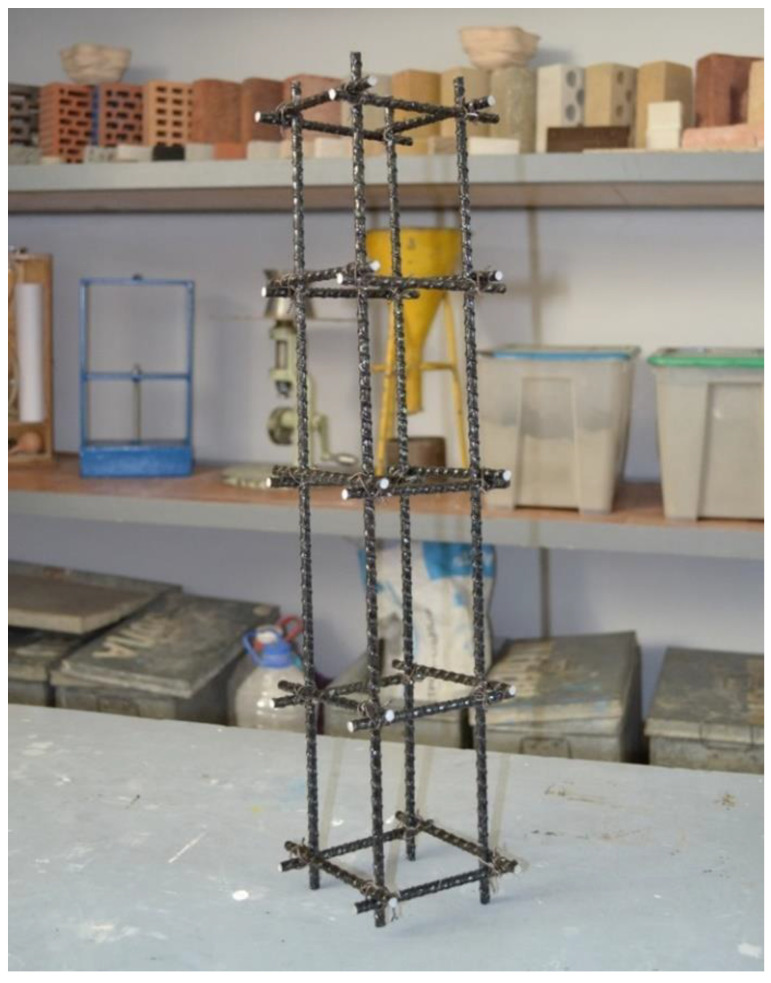
Photo of a frame made of polymer composite reinforcement.

A diagram of the reinforcement of the prototypes is shown in Figure 1, and a photograph of the frame made of polymer composite reinforcement is shown in Figure 2. The main characteristics of the prototypes are presented in Table 8.

**Figure 3:**
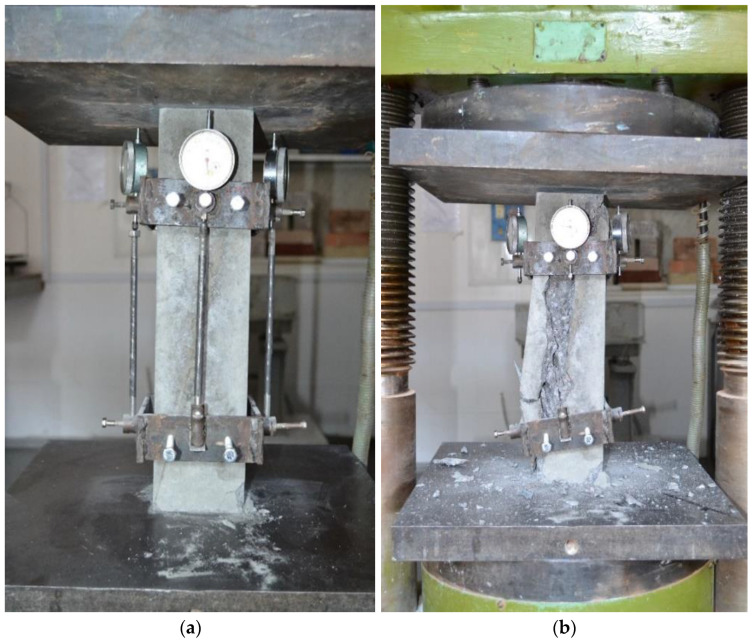
The collapse of the sample column during the test: (**a**) moment at the beginning; (**b**) final stage.

**Figure 4:**
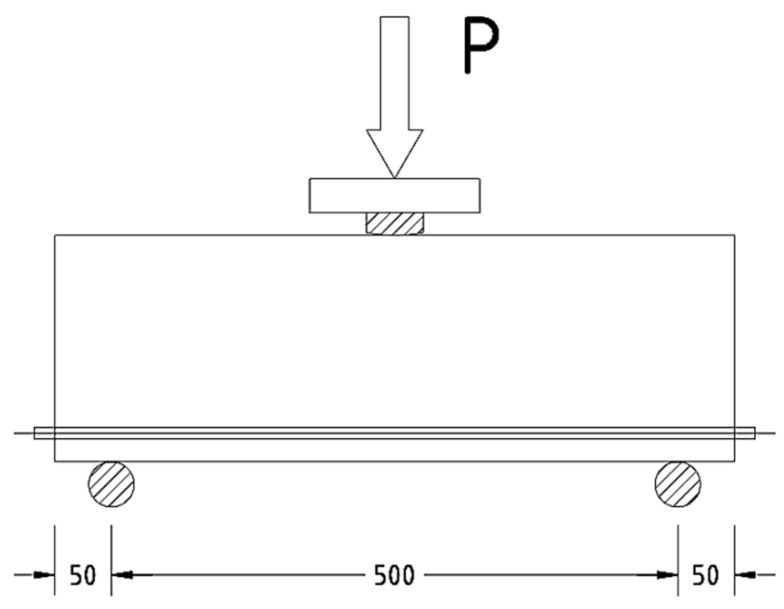
The design scheme of the bending test.

**Figure 5:**
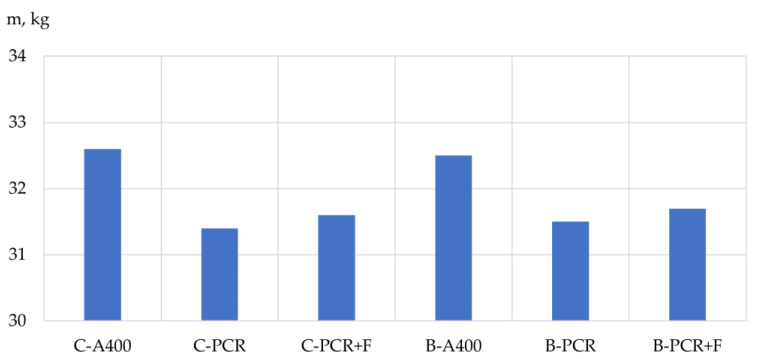
Dependence of the mass of the prototype columns and specimen beams on the type of reinforcement used: C-A400 is the sample column reinforced with steel reinforcement of class A400; C-PCR is a sample column reinforced with polymer composite reinforcement; C-PCR+F is a sample column reinforced with polymer composite reinforcement and glass fiber; B-A400 is a sample beam reinforced with steel reinforcement class A400; B-PCR is a sample beam reinforced with polymer composite reinforcement; B-PCR+F is a sample beam reinforced with polymer composite reinforcement and glass fiber.

The prototypes were tested at the age of 35 days from the date of manufacture. The prototypes were tested on a PGI-500 press. When testing prototype columns for compression, the load was applied step by step in 10–12 stages, with the same increments of longitudinal deformations that made it possible to follow the work of prototypes when the load increased to maximum, and then it decreased on the descending branch until destruction. After each stage of loading, the element was held for 10 min. At each stage, the readings of the devices were taken twice: immediately after increasing the load and after holding before the next stage of loading. Compression tests of specimens are shown in Figure 3 and Figure 4. The bending test of the prototypes was carried out according to the following design scheme: as beams (see Figure 5) lying on two hinged supports.

To determine the actual strength characteristics of concrete, simultaneously with the testing of each series of samples, tests of reference sample cubes were carried out. Compression tests of cubes were carried out in accordance with the requirements of GOST 10,180 “Concretes. Methods for strength determination using reference specimens” [34] (This standard complies with the basic regulations for the production and testing of concrete specimens given in the following European regional standards: EN 12390-1 “Testing hardened concrete–Part 1: Shape, dimensions and other requirements of specimens and moulds”; EN 12390-2 “Testing hardened concrete–Part 2: Making and curing specimens for strength tests”; EN 12390-3 “Testing hardened concrete–Part 3: Compressive strength of tests specimens”; EN 12390-4 “Testing hardened concrete–Part 4: Compressive strength-Specification for testing machines”; EN 12390-5 “Testing hardened concrete–Part 5: Flexural strength of tests specimens”; EN 12390-6 “Testing hardened concrete–Part 6: Tensile splitting strength of tests specimens”). The sample cubes were loaded to failure at a constant rate of load growth (0.6 ± 0.2) MPa/s.

In the bending test, the prototypes were loaded to failure at a constant rate of increase in the load (0.05 ± 0.01) MPa/s.

In tensile testing, the prism samples were loaded to failure at a constant rate of increase in the load (0.05 ± 0.01) MPa/s.

Tests of prisms for axial compression and axial tension were carried out at a constant rate of deformation to obtain the strength and deformation characteristics of concrete and its full deformation diagrams “σ–ε” with descending branches. The measurements of the concrete deformations of the test prisms were carried out by a chain of strain gauges with a side length of 50 mm and dial indicators with a graduation value of 0.001 mm. Axial compression and axial tension tests were carried out following the requirements of GOST 24,452 “Concretes. Methods of prismatic, compressive strength, modulus of elasticity and Poisson’s ratio determination” (interstate standard) [35].

When determining the prismatic strength of concrete, loading the specimen to a load level of 40 ± 5% was performed in steps equal to 10% of the expected breaking load, keeping within each step the loading rate 0.6 ± 0.2 MPa/s. At each stage, the load was held for 4–5 min, and readings were recorded by the instruments at the beginning and at the end of the load stage. At a load level equal to 40 ± 5%, the devices were removed from the prism samples, after which further loading of the sample should be performed continuously at a constant load rate 0.6 ± 0.2 MPa/s.

The study of the microstructure was carried out on a VEGA II LMU scanning electron microscope (Tescan, Brno, Czech Republic) at an accelerating voltage of 20 kV.

Images were obtained using SE and BSE detectors. The SE (secondary electron) detector provides information on the surface morphology of the sample. The BSE detector (reflected or backscattered electrons) provides information on the phase and chemical inhomogeneity of the material (phases and areas with a higher average atomic weight are colored in lighter shades).

The surface of the samples was sputtered with metal using an Emitech sputtering device.

## 3. Results

**Figure 6:**
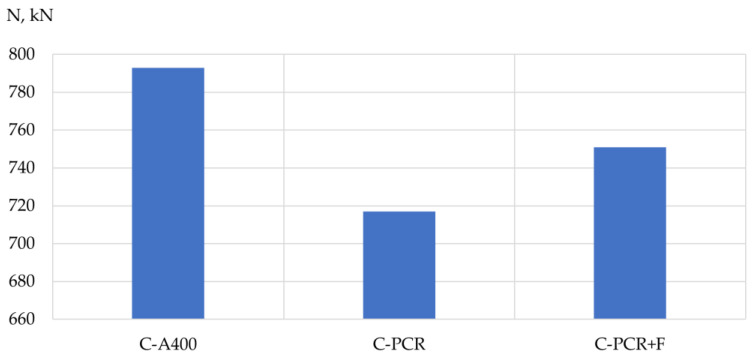
Dependence of the bearing capacity of the column specimens on the type of reinforcement used: C-A400 is a column specimen reinforced with A400 steel reinforcement; C-PCR is a column specimen reinforced with polymer composite reinforcement; C-PCR+F is a column specimen reinforced with polymer composite reinforcement and glass fiber.

**Figure 7:**
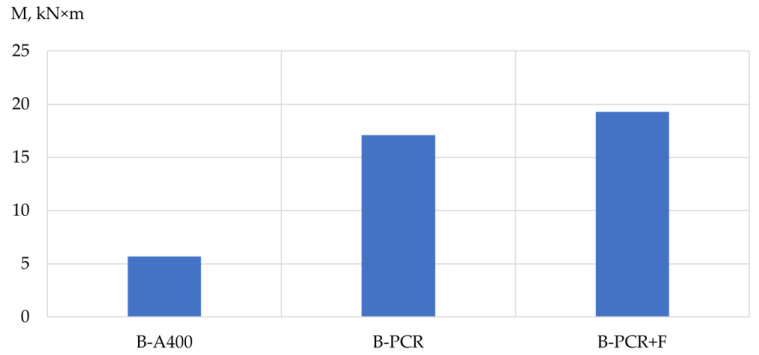
Dependence of the bearing capacity of specimen beams on the type of reinforcement used: B-A400 is a specimen beam reinforced with steel reinforcement of class A400; B-PCR is a sample beam reinforced with polymer composite reinforcement; B-PCR+F is a sample beam reinforced with polymer composite reinforcement and glass fiber.

**Figure 8:**
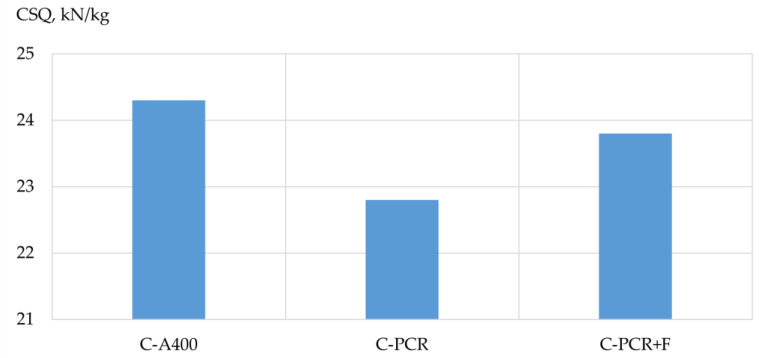
Dependence of the structural quality factor of the sample columns on the type of reinforcement used: C-A400 is a sample column reinforced with steel reinforcement of class A400; C-PCR is a sample-column reinforced with polymer composite reinforcement; C-PCR+F is a sample-column reinforced with polymer composite reinforcement and glass fiber.

**Figure 9:**
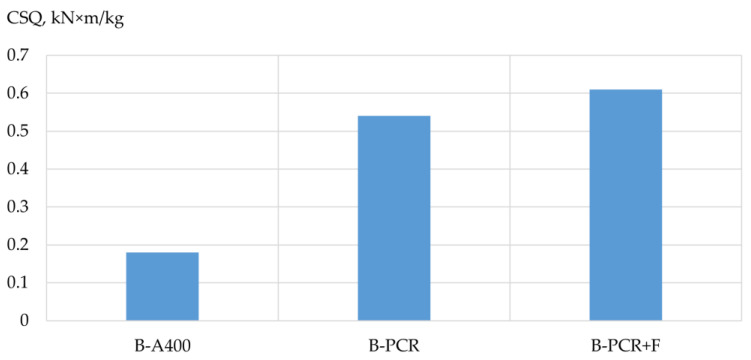
Dependence of the structural quality factor of sample beams on the type of reinforcement used: B-A400 is a sample beam reinforced with steel reinforcement of class A400; B-PCR is a sample beam reinforced with polymer composite reinforcement; B-PCR+F is a sample beam reinforced with polymer composite reinforcement and glass fiber.

**Figure 10:**
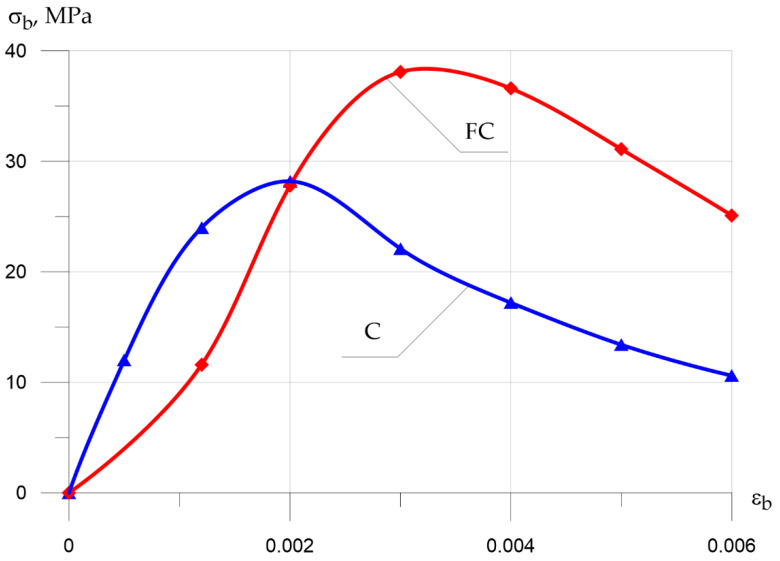
Stress–strain diagram for concrete compression (C, concrete; FC, fiber concrete).

Based on the results of the experimental studies, the bearing capacity, mass and structural quality factor (CSQ) [33] of the prototypes’ columns and beams were analyzed depending on the type of reinforcement used. The results are shown in Table 9, Table 10 and Table 11 and Figure 5, Figure 6, Figure 7, Figure 8, Figure 9 and Figure 10.

The CSQ was calculated using the following formulas:

- For sample columns:
  (1) $$CSQ_{c}=\frac{N}{m_{c}}$$
  where *N* is the bearing capacity of the column specimens, kN; $m_{c}$ is the mass of the column sample, kg.
- For sample beams:
  (2) $$CSQ_{b}=\frac{M}{m_{b}}$$
  where *M* is the bearing capacity of the sample beam, kN × m; $m_{b}$ is the mass of the sample beam, kg.

In the analysis performed, the indices of the column specimen reinforced with a steel reinforcement frame were taken as the starting point of reference.

The influence of the type of reinforcement used on the mass of the prototype columns and beams. According to the experimental data obtained, the samples reinforced with a frame made of steel reinforcement (up to 5% more mass of concrete samples with PCR) had the highest mass. As for the mass of samples reinforced with a frame made of polymer composite reinforcement without adding fiber and with the addition of glass fiber in the amount of 4% of the cement mass, their masses differed slightly, which is logical and is explained by the content of dispersed fiber.

The influence of the type of reinforcement used on the bearing capacity of the prototype columns. According to the experimental data obtained, the highest bearing capacity was possessed by a column reinforced with a frame made of steel reinforcement, and the value of its bearing capacity was 10% higher than that of a column reinforced with polymer composite reinforcement. As for the column reinforced with a frame made of polymer composite reinforcement with the addition of fiber, its bearing capacity increased by 5% in comparison with a column reinforced with a frame made of polymer composite reinforcement without adding fiber.

The influence of the type of reinforcement used on the bearing capacity of the prototype beams. According to the experimental data obtained, the highest bearing capacity was possessed by a beam reinforced with a frame made of polymer composite reinforcement with the addition of fiber. The value of its bearing capacity was 3.4 times higher than that of a beam reinforced with a frame made of steel reinforcement. As for the beam reinforced with a frame made of polymer composite reinforcement without the addition of fiber, its bearing capacity was three times higher in comparison with a beam reinforced with a frame made of steel reinforcement.

Influence of the type of reinforcement used on the structural quality factor of the prototype columns. According to the obtained experimental data, the column reinforced with a frame made of polymer composite reinforcement without the addition of fiber had the lowest coefficient of structural quality. As for the coefficient of structural quality of a column reinforced with a frame made of steel reinforcement and a column reinforced with a frame of polymer composite reinforcement with the addition of fiber, the values of their structural quality coefficients had insignificant differences.

Influence of the type of reinforcement used on the structural quality factor of the prototype beams. According to the experimental data obtained, the highest coefficient of structural quality was possessed by the beam reinforced with a frame made of polymer composite reinforcement with the addition of fiber. The values of its structural quality coefficient were 3.4 times higher in comparison with the coefficient of the structural quality of a beam reinforced with frames made of steel reinforcement. As for a beam reinforced with a polymer composite reinforcement frame without adding fiber, the values of its structural quality factor were three times higher.

**Figure 11:**
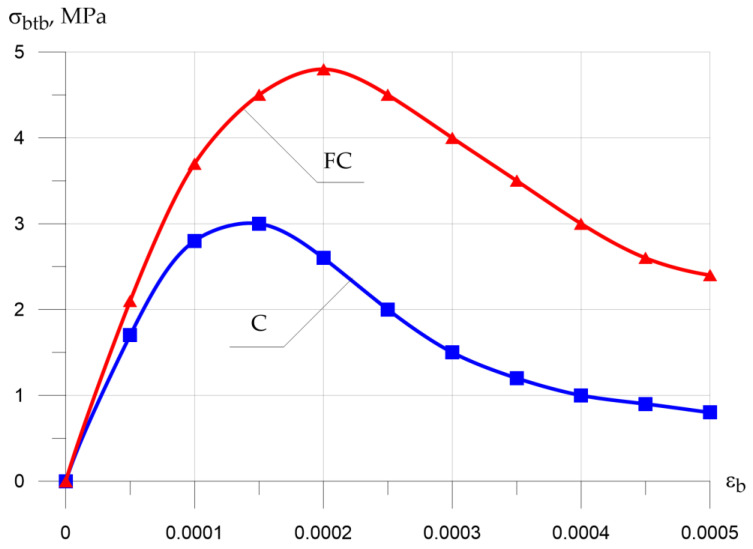
Stress–strain diagram for tensile concrete (C, concrete; FC, fiber-reinforced concrete).

Figure 10 and Figure 11 show the “$\sigma_{b}-\varepsilon_{b}$” compression and “$\sigma_{btb}-\varepsilon_{btb}$” tension diagrams for concrete and fiber-reinforced concrete.

Analysis of the deformation diagrams obtained from the experimental data revealed the following characteristic features: in fiber-reinforced concrete, in comparison with concrete, the top of the diagrams fits up and to the right, which is explained by an increase in mechanical strength and giving the material a more viscous character of destruction and an increase in the tested ultimate deformations until the destruction of the sample due to the dispersed micro-reinforcement with glass fiber.

For polymer composite reinforcement, the relative elongation is directly proportional to the tensile load up to failure, in contrast to steel, which has a zone of elastic work, a yield point, a zone of self-strengthening, and rupture. This means that, when operating under load, the deflections of structures reinforced with polymer composite reinforcement will increase uniformly, up to destruction, in proportion to the increase in external load, in contrast to structures reinforced with steel reinforcement. A feature of the operation of steel reinforcement is the presence of a stage when deflections grow without increasing the external load at the moment of reaching stresses corresponding to the yield point, which is not observed in polymer composite reinforcement [36,37,38].

**Figure 12:**
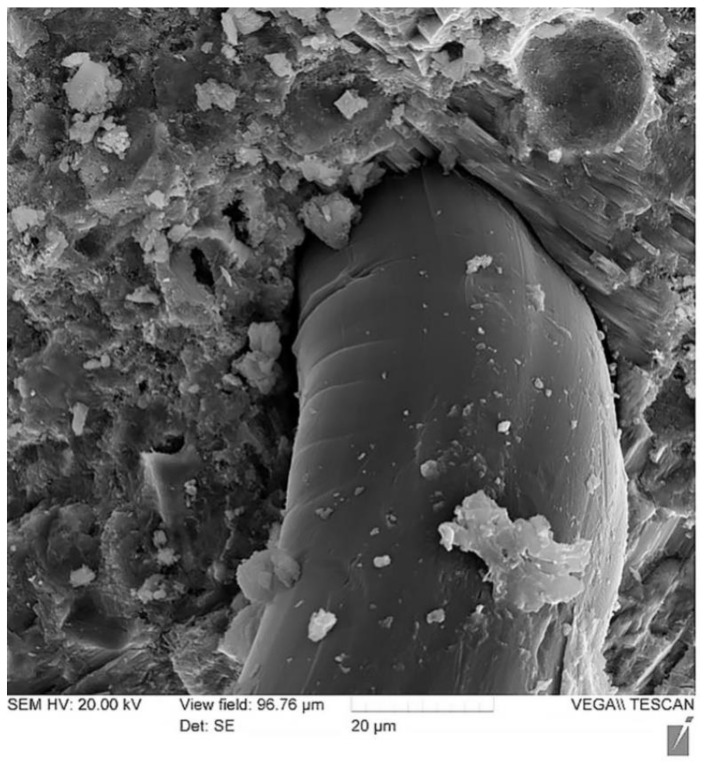
The nature of glass fiber deformation.

The image in Figure 12 shows the nature of glass fiber deformation with a 2000-fold magnification performed on a TESCAN VEGA II LMU scanning electron microscope at an accelerating voltage of 20 kV.

This image makes it possible to establish that the fiber undergoes deformation when the sample is broken and pulled out of the body of the cement stone, which is noticeable in the image and is expressed in the change in the thickness of the fiber and characteristic bends visible in the micrograph. Thus, it is obvious that when the sample is deformed, the fiber, in turn, undergoes its own deformation, which, however, is not so significant, and when fractured, it is the fiber that creates the so-called damping effect and imparts a viscous character of fracture to the cement matrix. Therefore, using these fibers makes it possible to smooth out the “explosive” nature of the destruction of a rather brittle material—concrete, especially high-strength concrete.

This SEM image is used to support the hypothesis that the fibers tend to break rather than pull out (which is often a problem with inappropriately used fibers). Observing the ongoing fiber deformation, it can be concluded that the applied fiber was well anchored in the body of the cement matrix and, thus, the selected recipe approach is correct.

This can explain and substantiate the need for additional fiber reinforcement in a rational range to give high-strength concrete with high-strength reinforcement a more viscous nature of destruction. Thus, at the micro-level, with the help of a microscope, our working hypothesis is confirmed, and the results of experimental studies of the mechanical properties of the obtained sample are supplemented.

**Figure 13:**
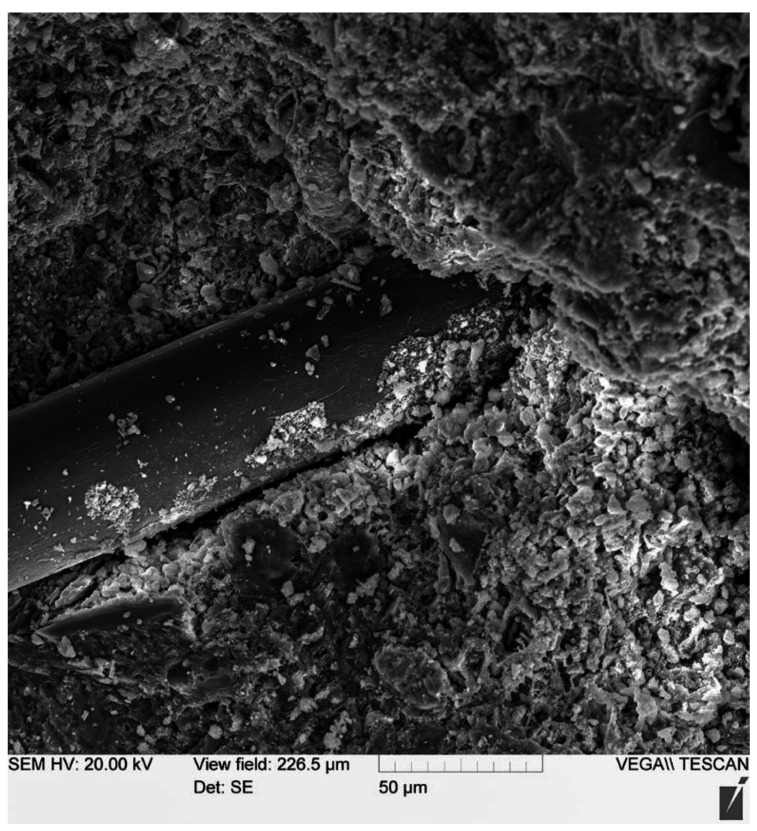
The contact zone and the nature of the interaction of the fiber with the cement matrix.

The photo (Figure 13) shows the contact zone and the nature of the interaction of the fiber with the cement matrix.

The fiber in this case is a filler and the cement stone is a matrix. The photograph clearly shows the contact zone between them. At the micro-level, the fiber is also exposed to the adhesion of particles of the hydration products of the cement stone, which suggests a good degree of adhesion between the fiber and the cement stone. Thus, the fiber, which has both the function of a damper and imparting a soft tough character of destruction, is also a fairly integrated component that is not rejected by the cement stone, despite its foreign character in the concrete body, and it confirms a high level of adhesion as well as anchoring in the concrete body.

In combination with rod reinforcement with polymer composite reinforcement, the nature of the adhesion of which with a cement stone can be seen with the naked eye due to the fact of its large size, this image (Figure 13) confirms the combination of the cement stone as a matrix and a concrete body as a conglomerate of sufficiently good macro-reinforcing elements, i.e., polymer composite reinforcement rods and micro-reinforcing components of dispersed glass fibers.

## 4. Discussion

Comparing our study with studies previously conducted by other authors, it should be noted that there are differences in the methodological approach.

In the classical approach, authors working with compressed and bending reinforced concrete elements phenomenologically set the reinforcement parameters, usually taking into account only traditional types of steel, differing in diameter and number of rods.

If we are talking about the study of fiber-reinforced concrete, then, as a rule, in this case, the authors phenomenologically set the classical parameters of dispersed reinforcement: the percentage of reinforcement, the length of the fibers, and the choice of material is determined by the accumulated experience.

Our phenomenological approach was based on the hypothesis that high-strength reinforcement, among other things, does not use its resource to the full due to the fact that concrete takes on a large share of the bearing capacity in reinforced concrete elements. Therefore, our goal in the formation of our phenomenological approach, in contrast to other authors, was the idea to increase the bearing capacity while reducing the weight and, at the same time, allowing the structure of the concrete stone to obtain the required useful deformability due to the dispersed reinforcement and hardening of the reinforced concrete element at the micro level.

Thus, we are considering our phenomenological model, which is based on two assumptions.

It is possible to simultaneously reinforce concrete elements both with rods, that is, at the macro level and with dispersed fiber, that is, at the micro-level.

In this case, an important factor is the material for the manufacture of fibers and reinforcing bars.

Thus, our research is a pivotal basis for the subsequent development of research in the direction of numerical modeling, the operation of rods from various materials in combination with dispersed reinforcing fibers, also made from various materials.

As part of a comparative analysis of our research with research by other authors, two main criteria can be identified:

- Comparison by type of reinforcing elements;
- Comparison by type of stress–strain state.

None of the analyzed works [22,23,24,25,26,27,28,29,30,31] considered combined reinforcement with dispersed fiber in combination with polymer composite reinforcement. In terms of the investigated stress–strain state, the authors of these works considered the absolute indicators: the strength of concrete for various types of loads and the reinforced element’s bearing capacity.

Thus, the scientific novelty of the study is the application of combined reinforcement of heavy concrete with polymer composite reinforcement in combination with fiber. Such studies have not previously been conducted. It resulted that the technology of combined concrete reinforcement with new lightweight types of reinforcing components provides a synergistic effect, significantly bringing the characteristics of such an element to the characteristics of a traditional reinforced concrete analogue. At the same time, the structure’s weight was reduced considerably, opening up the prospects for the use of such lightweight and durable elements in the construction of new high-rise and large-span buildings and structures.

Several factors can explain the resulting synergistic effect.

Firstly, the main reason for the increase in the coefficient of constructive quality (up to 240%) of the combined reinforced element is a reduction in its mass (up to 5% with the adopted reinforcement scheme) compared to the reinforced concrete analogue. The mass of the element reinforced only with polymer composite reinforcement was not that much inferior to the mass of the combined reinforced element (up to 0.7%).

Secondly, the strength characteristics of the concrete itself, that is, the element that receives compressive loads, increased significantly. Thus, concrete reinforced with dispersed fiber acquired greater structural strength under central compression, and the fragile nature of its destruction was prevented.

Thirdly, the additional fiber reinforcement of the bending element allowed the concrete to unload to a greater extent directly on the polymer composite reinforcement, which alone carries this function for simple types of reinforcement, only polymer composite or steel reinforcement.

Fourthly, due to the proposed recipe constructive solutions, an increase in the bearing capacity of the combined reinforced element was achieved up to 13% in relation to concrete reinforced only with PCR rods.

Additional evidence confirming the hypotheses about the synergistic effect was the SEM analysis, the plotted stress–strain diagrams for the compression and tension of concrete, the physical experiments with the determination of absolute indicators and the calculations of the relative indicators.

All this ultimately leads to a sharp increase in the coefficient of the structural quality of the reinforced element.

## 5. Conclusions

Based on the performed theoretical review and experimental research results, the following conclusions can be drawn.

Polymer composite reinforcement and concrete based on it, with the correct design and technological and recipe solutions, can approach the same performance to those obtained when using steel reinforcement and, at the same time, according to the studied indicators of the mass of the structure, its bearing capacity and the coefficient of structural quality. It was revealed that polymer composite reinforcement is still inferior to the used steel reinforcement in terms of bearing capacity in its pure form. Still, it significantly wins in terms of the indicator “weight constructions”. In quantitative terms, this is expressed as follows: the CSQ values of a beam reinforced with a PCR frame with the addition of glass fiber were 3.4 times higher in comparison with the CSQ of a beam reinforced with steel reinforcement frames, and the CSQ values of a beam reinforced with a PCR frame without adding fiber three times higher.

In this regard, in modern conditions of dense urban development and engineering-geological conditions in which they are forced to erect high-rise and large-span buildings and structures, this characteristic will be a great advantage. However, also according to the results of a theoretical review and experimental studies, it was revealed that an additional recipe, technological or constructive solutions are needed when reinforcing elements made of concrete. This solution turned out to be the combined reinforcement of heavy concrete with polymer composite reinforcement and dispersed fiber. Thus, in a qualitative aspect, the combined reinforcement had a significant effect, and when applying such a solution, it is possible to approximate the characteristics of polymer composite reinforcement and concrete based on it to those of traditional reinforced concrete.

In quantitative terms, this is expressed as follows: reinforcement with polymer composite reinforcement according to the previously presented scheme and the percentage of fiber reinforcement was 4% of the cement mass. Under this condition, the coefficient of the constructive quality indicator was almost identical to the traditional reinforced concrete.

The SEM analysis performed while observing the fiber deformation revealed that the applied fiber was well anchored in the body of the cement matrix and, thus, the selected recipe approach is correct.

Therefore, the resulting developments can be recommended in the practice of design and technology and construction of buildings and structures.

## Figures and Tables

**Table 1:** Characteristics of Portland cement PC 500 D0.

Grinding Fineness, by Specific Surface, cm^2^/g	Normal Density of Cement Paste,%	Setting Time of Cement Paste, min	Flexural Strength, MPa (at the Age of 28 Days)	Compressive Strength, MPa (at the Age of 28 Days)
3179	27.7	start—145 finish—250	8.1	52.5

**Table 2:** Mineralogical and chemical composition of Portland cement.

Mineralogical Composition, %				Chemical Composition, %					
C_3_S	C_2_S	C_3_A	C_4_AF	MgO	SO_3_	Na_2_O + K_2_O	CaO	SiO_2_	Lost on Ignition
68.8	10.3	8.9	11.3	0.9	2.34	0.75	50.1	14.0	3.56

**Table 3:** Physical and mechanical characteristics of coarse aggregate.

Bulk Density, kg/m^3^	Dust and Clay Particles, %	Crushing According to GOST 8269.0 [32], %	Grain Density, g/cm^3^	Voidness, %
1380	0.71	12.3	2.57	47

**Table 4:** Physical and mechanical characteristics of fine aggregate.

Bulk Density, kg/m^3^	Dust and Clay Particles, %	Size Module	Grain Density, g/cm^3^	Voidness, %
1480	0.65	2.57	2.61	43.3

**Table 5:** Comparative characteristics of metal and composite reinforcement.

Characteristics	Steel Reinforcement A400	Polymer Composite Reinforcement
Material	Steel	Glass roving bonded with epoxy resin
Ultimate tensile strength, MPa	340	1100
Elastic modulus, GPa	200	55
Elongation, %	25	2.2
Behavior under load (stress–strain relationship)	Curved line with yield line under load	A straight line with linear elastic dependence under load to failure
Density, g/cm^3^	7.0	1.5
Corrosion resistance	Corrodes, releasing rust products	Corrosion-resistant material of the first group of chemical resistance including to the alkaline environment of concrete

**Table 6:** Physical and mechanical characteristics of glass fiber.

Density, g/cm^3^	Tensile Strength, GPa	Elastic Modulus, GPa	Fiber Length, mm	Elongation, %
2.6	1.8	70	12	1.5

**Table 7:** Parameters of the composition of the concrete mixture.

Indicator Title	Cement, kg/m^3^	Water, L/m^3^	Crushed Stone, kg/m^3^	Sand, kg/m^3^	ρ_cm_, kg/m^3^
Indicator value	375	210	1028	701	2314

**Table 8:** Characteristics of prototypes.

Prototype Code	Sizes, mm			Consumption of Materials		
	Length	Width	Height	The Concrete Mixture, L	Reinforcement, kg	Fiber, g
C-A400	600	150	150	14.1	0.986	-
B-A400	600	150	150	14.0	0.979	-
C-PCR	600	150	150	14.2	0.193	-
B-PCR	600	150	150	14.1	0.201	-
C-PCR+F	600	150	150	14.0	0.194	210
B-PCR+F	600	150	150	14.0	0.197	210

C, column; B, beam sample; A400, a class of steel reinforcement; PCR, polymer composition reinforcement; F, glass fiber.

**Table 9:** Test results of control sample cubes.

Series Number	Compressive Strength, MPa	
	Samples	Average in Series
1	42.4	43.7
	43.9	
	44.9	
2	45.8	44.1
	43.1	
	43.5	
3	46.8	42.9
	41.5	
	40.5	

**Table 10:** The results of determining the structural characteristics of the sample columns.

Prototype Code	Mass, kg	Bearing Capacity, kN	$CSQ_{c},\;\mathrm{kN}/\mathrm{kg}$
C-A400	32.6	793	24.3
C-PCR	31.4	717	22.8
C-PCR+F	31.6	751	23.8

C, sample column; A400, a class of steel reinforcement; PCR, polymer composite reinforcement; F, glass fiber.

**Table 11:** The results of determining the structural characteristics of specimen beams.

Prototype Code	Mass, kg	Bearing Capacity, kN × m	$CSQ_{c},\;\mathrm{kN}\;\times \;m/\mathrm{kg}$
B-A400	32.5	5.7	0.18
B-PCR	31.5	17.1	0.54
B-PCR+F	31.7	19.3	0.61

B, beam sample.

## Data Availability

Data sharing not applicable.
